# Navigating Solid Tumor Heterogeneity: The Promise and Challenges of Antibody-Drug Conjugates

**DOI:** 10.47248/chp2502040017

**Published:** 2025-10-15

**Authors:** Ashley A. Duhon, Karen McLean

**Affiliations:** 1. Department of Gynecologic Oncology, Roswell Park Comprehensive Cancer Center, Buffalo, NY 14203, USA; 2. Department of Pharmacology & Therapeutics, Roswell Park Comprehensive Cancer Center, Buffalo, NY 14203, USA

**Keywords:** antibody drug conjugate, payload, tumor heterogeneity, tumor microenvironment, resistance, drug development, combination therapy

## Abstract

Antibody drug conjugates (ADCs) are changing the landscape of cancer therapy. These agents contain an antibody directed at a tumor cell surface antigen linked to a cytotoxic payload that is released following complex internalization and processing by the lysosome. To date, seven ADCs have been approved by the Federal Drug Administration for the treatment of solid tumors and an additional seven ADCs are approved in hematologic malignancies; because of the unique aspects of solid tumor therapy, this review will focus specifically on ADCs for solid malignancies. Review of the design of these solid tumor ADCs highlights the successful evolution of ADC treatment to date, including selection of antigen target, chemical linker features, and payload. In this review, we focus on how spatial and temporal intratumoral heterogeneity uniquely limits durable efficacy of ADC therapy. We consider strategies to overcome these hurdles, including improved characterization of clinical samples for optimal ADC selection, improvements in ADC design, and combinatorial therapy. These preclinical and clinical efforts seek to overcome the challenges of tumor heterogeneity to improve ADC options and outcomes in the treatment of solid tumor malignancies.

## 1. Introduction

**Figure 1.**
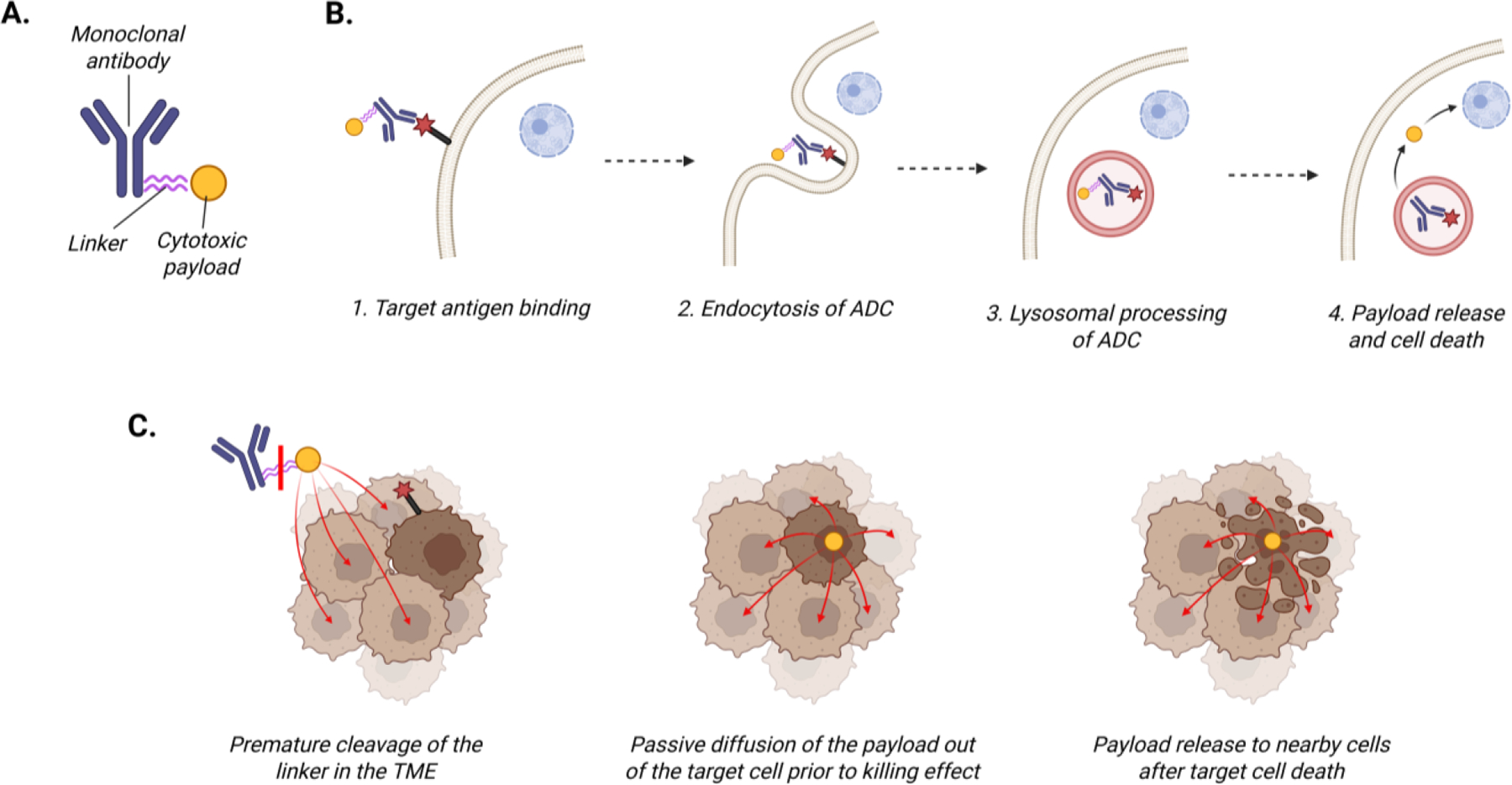
Fundamental features of ADCs. (A) Structure of ADCs. (B) Mechanism of action. (C) Bystander effect, due to multiple mechanisms, including ADC linker cleavage in the cytoplasm, payload diffusion out of the target cell, or payload release after target cell death. This figure was created using Biorender.com.

Approximately 40% of people in the United States will be diagnosed with cancer in their lifetime, with a projected two million new diagnoses and over 600,000 cancer-related deaths in the country in 2025 [1]. Cytotoxic chemotherapy remains the standard of care for the treatment of many cancers; however, the development of resistance to standard therapies and adverse effects remain issues in establishing durable therapeutic responses. Antibody drug conjugates (ADCs) are a treatment option that harnesses mechanisms of both immunotherapy and traditional chemotherapy by delivering cytotoxic treatments directly to tumor cells. ADCs are composed of a tumor-directed antibody with a cytotoxic agent linked to it (Figure 1A). For an ADC to exert its antitumor effects, the antibody binds to the target antigen on the surface of tumor cells (Figure 1B). The ADC complex is then internalized into the cell through endocytosis and undergoes cellular processing. The nature of the linker determines the ADC’s stability in plasma prior to being internalized by the cancer cells. The cytotoxic payload is subsequently released into the cytoplasm of the cancer cell leading to cell death [2]. Currently employed payloads are cytotoxic drugs that exert their killing effect on the cancer cell through either induction of DNA damage or inhibition of microtubule function.

Three distinct generations of ADCs have been described, each widening the therapeutic window of the ADC. First generation ADCs utilized murine antibodies that induced high secondary immunogenicity and unstable linkers that often resulted in inconsistent and early payload release in the serum prior to delivery to the target cell. An example of a first generation ADC is gemtuzumab ozogamicin, the first FDA-approved ADC with an indication for use in patients with acute myeloid leukemia. The second generation of ADCs improved on initial limitations with the incorporation of antibody modifications, more stable linkers, and higher levels of cytotoxic drug conjugation, allowing for more focused drug delivery with less toxicity. Trastuzumab emtansine is the only second generation ADC approved for solid tumors (Table 1). The remaining currently approved ADCs for solid tumors are classified as third generation (Table 1) and are characterized by additional structural changes to allow even more stabilized antibody-to-payload linkage while minimizing interference with antibody-target antigen binding [3–5].

Since the first ADC was approved in 2000, continued preclinical research and clinical translation have extended the use of ADCs for both liquid and solid tumor cancers. There are now fourteen ADCs approved by the Food and Drug Administration (FDA) across tumor types including seven in solid tumors, with more than 100 ADCs in various stages of clinical development [6]. Much remains to be elucidated with respect to optimizing ADC efficacy while minimizing both the development of resistance and treatment-related toxicities. As the field has advanced, it has become apparent that tumor heterogeneity is a crucial factor impacting ADC treatment efficacy. Furthermore, our evolving understanding of bystander effect, in which the cytotoxic payload reaches nearby cells that do not necessarily express the target antigen (Figure 1C), is impacting ADC design and clinical use [7]. The purpose of this review is to discuss the current state of ADC development in solid tumors, with a focus on agent design and the impact of tumor heterogeneity. We will present strategies to robustly assess tumor heterogeneity prior to ADC therapy, ADC design approaches to minimize the impact of heterogeneity on therapeutic efficacy, and the role of combinatorial therapies. The integration of these perspectives into preclinical and clinical ADC development has the potential to significantly advance the therapeutic benefit for patients as the landscape of solid tumor therapy continues to evolve.

## 2. Insights from the Evolution of Clinically Approved ADCs

Detailed consideration of currently approved ADCs provides insights into the fundamental principles of ADC design for successful antitumor effects. There are seven ADCs currently FDA-approved for solid tumors with five unique target antigens (Table 1). These are trastuzumab emtansine (T-DM1), trastuzumab deruxtecan (T-DXd), sacituzumab govitecan (SG), datopotamab deruxtecan (Dato-DXd), enfortumab vedotin (EV), tisotumab vedotin (TV) and mirvetuximab soravtansine (MIRV) [6,8].

The first FDA-approved ADC for clinical use in a solid tumor was trastuzumab emtansine (T-DM1), which targets human epidermal growth factor receptor 2 (HER2/ERBB2). HER2 overexpression is a known feature of a subset of breast cancers, with overexpression or activation of the ERBB2 receptor tyrosine kinase (RTK) leading to pro-tumorigenic cell signaling [9] that has been successfully clinically targeted with the monoclonal antibody trastuzumab [10], making it an ideal target for the first ADC for solid tumors. The structure of T-DM1 highlights early design features of ADCs. It utilizes a humanized monoclonal antibody attached to the cytotoxic payload DM1 via a non-cleavable linker. DM1 is a maytansinoid compound that functions to bind tubulin and inhibit microtubule assembly during cell division. Because T-DM1 utilizes a non-cleavable linker, release of the cytotoxic payload is dependent on degradation of the antibody component within the target cell lysosome while the whole linker remains attached to the payload. The payload can then exert its effects in the cell in which it was internalized, but bystander effect to neighboring cells is limited because the linker-payload conjugate cannot diffuse across the cell membrane due to its hydrophilic nature and, therefore, is not released extracellularly until cell death occurs. Multiple clinical trials have demonstrated the effectiveness of T-DM1 in HER2 positive (HER2+) breast cancers. Specific approval indications include treatment of early HER2+ breast cancer with residual disease after neoadjuvant treatment with trastuzumab and taxane therapy [11] and metastatic HER2+ breast cancer previously treated with trastuzumab and a taxane [12,13].

Trastuzumab deruxtecan (T-DXd) also targets HER2/ERBB2 but is functionally different from T-DM1 in two key features: it employs the cytotoxic payload DXd attached via a linker that is cleaved by lysosomal proteases. DXd is a topoisomerase I (TOPI) inhibitor that traps TOPI complexes during the relaxation of DNA supercoiling that takes place for DNA. This results in the subsequent induction of apoptosis. T-DXd has more potential bystander effect than T-DM1 due to the cleavable linker that provides the opportunity for the intact payload to be released and undergo passive diffusion across the cell membrane after the linker is cleaved but before the death of the initial cell in which the ADC was internalized [6,8]. It is approved for unresectable HER2+ breast cancer in patients who have received two or more anti-HER2 therapies [14], HER2-low or -ultralow breast cancer [15], and HER2+ non-small cell lung cancer (NSCLC) [16] and gastric adenocarcinoma [17].

Sacituzumab govitecan (SG) and datopotamab deruxtecan (Dato-DXd) both target Trop2 (also known as trophoblast cell surface antigen 2, tumor-associated calcium signal transducer-2/TACSTD2). Trop2 is a transmembrane glycoprotein that functions in cell signaling for proliferation, invasion, and survival via pathways including the mitogen-activated protein kinase (MAPK) and phosphatidylinositol 3-kinase/protein kinase B (PI3K/AKT) pathways. Trop2 overexpression has been reported in multiple tumor types [18]. SG consists of a humanized monoclonal antibody attached to the payload SN-38 through a pH-sensitive hydrolysable cleavable linker CL2. SN-38 is TOPI inhibitor that induces cellular apoptosis through DNA damage. Dato-DXd utilizes the same DXd linker-payload as is used in T-DXd. The anti-Trop2 antibody is attached to the DXd payload via an enzyme-cleavable, tetrapeptide linker. The peptide-based, enzyme-cleavable linker utilized in Dato-DXd is more stable in serum than the linker in SG, thus decreasing the likelihood of extracellular payload release prior to reaching the target cell [19,20]. SG is approved for use in metastatic triple negative breast cancer [21] and as second-line therapy for urothelial cancer patients who have already received platinum and immune checkpoint inhibitor based-therapies [22]. Dato-DXd is approved for use in unresectable or metastatic hormone receptor-positive, HER2-negative breast cancer in patients who have received prior endocrine-based therapy and chemotherapy [23], and for patients with locally advanced or metastatic epidermal growth factor receptor (EGFR)-mutated NSCLC who have received prior EGFR-targeted therapies [24,25].

Three additional ADCs have been approved for solid tumors, each with a unique antigen target. Enfortumab vedotin (EV) is a third generation ADC that utilizes a human monoclonal antibody targeting Nectin-4 (PVRL4). Nectin-4 is a type I transmembrane protein and tumor-associated antigen that has been shown to play a role in signaling for tumor cell proliferation, differentiation, and invasion via the PI3K/AKT pathway. It is overexpressed in most urothelial carcinomas. In EV, the anti-Nectin-4 antibody is linked to the payload monomethyl auristatin E (MMAE) via the enzyme-cleavable linker mc-VC-PABC. MMAE is a cytotoxic drug within the auristatin family and blocks tubulin polymerization during cell division [26]. EV is approved for use in locally advanced or metastatic urothelial cancer [27].

Tisotumab vedotin (TV) uses a human monoclonal antibody targeting tissue factor (TF), also known as thrombospondin kinase, coagulation factor III, or CD142. TF is transmembrane glycoprotein that in normal tissues initiates the exogenous coagulation pathway for hemostasis. In tumor tissues, TF overexpression is associated with enhanced tumor growth, perhaps due to increased angiogenesis [28]. TV also utilizes MMAE as its payload with linkage by the enzyme-cleavable mc-VC-PABC, as with EV. TV is currently approved for recurrent cervical cancer [29].

Finally, mirvetuximab soravtansine (MIRV) utilizes a monoclonal antibody targeting folate receptor alpha (FRα, encoded by the *FOLR1* gene). FRα is a glycophosphatidylinositol (GPI)-anchored membrane protein that functions in the transport of folate into cells for DNA synthesis [30]. The anti-FRα antibody is attached to the DM4 payload via the enzyme-cleavable linker sulfo-SPDB. DM4 is a maytansinoid compound targeting microtubules, which is within the same family as the DM1 payload in T-DM1. As DM4 is linked to the antibody through a cleavable linker, it is less stable in serum with more potential for bystander effect to non-target cells when compared to T-DM1. MIRV is approved in recurrent, platinum-resistant ovarian cancer with greater than 75% expression of FRα [31].

These FDA-approved ADCs for solid tumors (Table 1) have several shared features that highlight successful ADC design for clinical translation. The antibody targets are all membrane bound proteins expressed on the surface of tumor cells. Furthermore, apart from FRα, all targets are transmembrane proteins with a cytosolic domain that mediates pro-tumorigenic cell signaling. The ADC payloads for all FDA-approved agents target either microtubules or topoisomerase I and are highly cytotoxic at low concentrations. Linking multiple payload molecules to a single antibody, termed the drug-to-antibody ratio (DAR), can also increase ADC efficacy. An additional factor that may improve the efficacy of a payload is the presence of modifiable functional groups for robust chemical conjugation that reduces premature payload release. For example, chemically modifying maytansine to add a thiol group yields DM1, and then a disulfide bond can be used to attach DM1 to the ADC linker. Improving the chemical stability of the ADC in the mildly acidic tumor microenvironment (average pH 6.5–7.0) may defer degradation of the linker and subsequent payload release until processing within the much more acidic environment of the intracellular lysosome (pH 4.5–5.0) [32–34]. Finally, most linkers are cleavable. A strength of cleavable linkers is the potential to increase bystander effect to facilitate killing of cells that do not express that ADC target antigen (Figure 1C); however, this may also increase treatment-related toxicity. Despite the treatment efficacy that has been demonstrated by the ADCs currently in clinical use and the improvement in outcomes that have come with subsequent generations of ADCs, clinical challenges remain with tumor response to treatment at baseline and over time. A key driver of both inherent resistance and lack of durable response is tumor heterogeneity.

## 3. Role of Tumor Heterogeneity in ADC Target Selection and Efficacy

**Figure 2.**
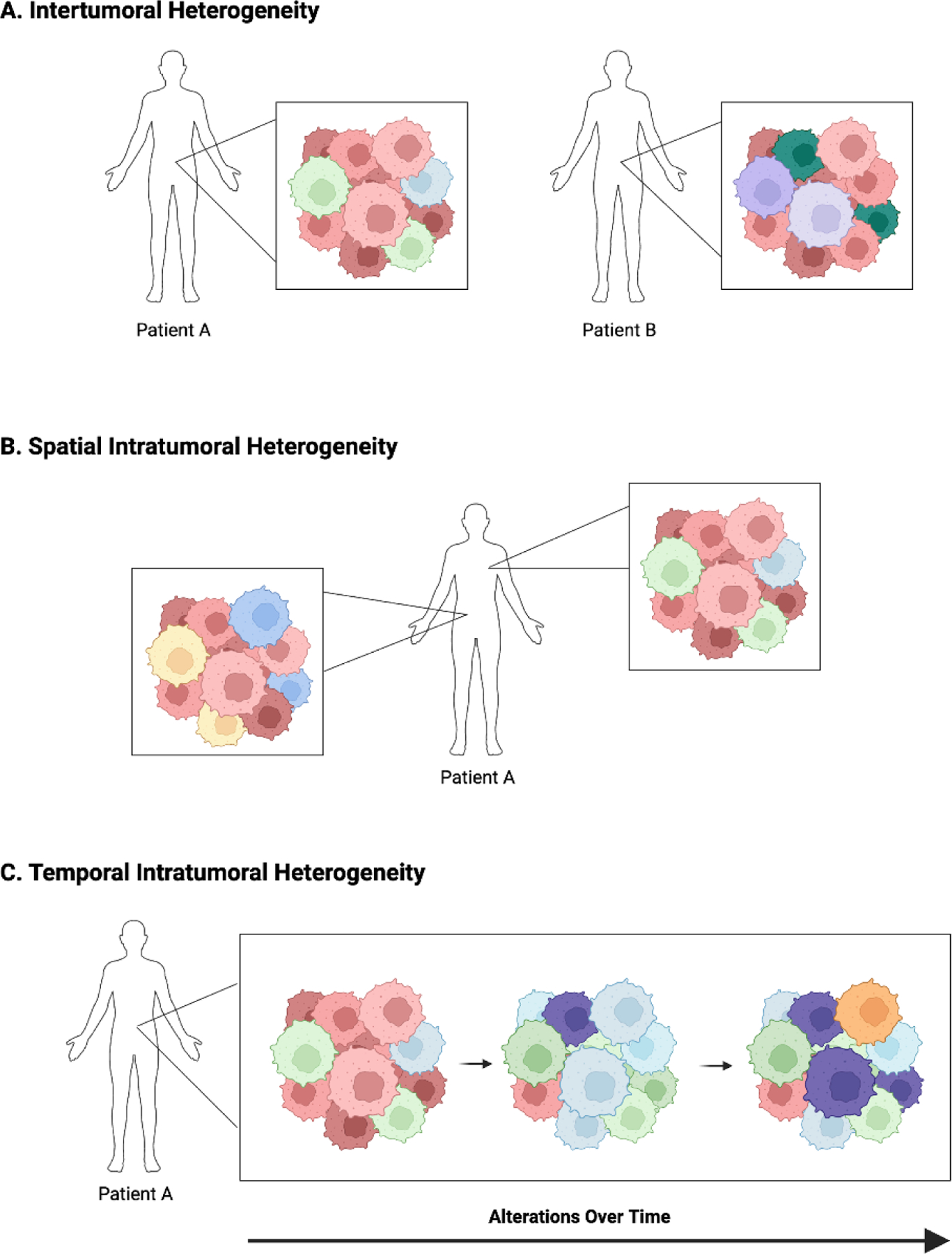
Types of Tumor Heterogeneity. (A) Intertumoral heterogeneity. (B) Spatial intratumoral heterogeneity. (C) Temporal intratumoral heterogeneity. This figure was created using Biorender.com.

Tumor heterogeneity is a hallmark of cancers (Figure 2), both during initial development and throughout treatment and progression, leading to constant tumor evolution over time [35,36]. These changes result in multiple different cancer cell populations within a single patient, each with unique genetic and proteomic signatures. This heterogeneity is referred to as *intra*tumoral heterogeneity and ultimately leads to both inherent and acquired tumor resistance to cancer-directed therapies [37–39]. Heterogeneity can also be described as *inter*tumoral, which refers to molecular differences in tumors of the same type between patients (Figure 2A). These differences are primarily due to patient-specific factors, either endogenous due to an individual’s genetics or exogenous due to external factors such as environmental influences [37]. While both types of heterogeneity present challenges in refining precision-based therapy for cancer at the population level, the remainder of this review will focus on intratumoral heterogeneity and its impact on ADC efficacy and resistance.

Intratumoral heterogeneity can be further defined as spatial or temporal. Spatial heterogeneity is used to refer to variations between the geographic regions within a single tumor or between the primary tumor and its metastatic sites (Figure 2B) [37]. Spatial differences in cell populations have been shown to exist even prior to completion of malignant transformation. Pre-malignant lesions in NSCLC [40], esophageal cancer [41], and melanoma [42] all demonstrate spatial heterogeneity. As malignant transformation progresses and genomic instability increases, new subclonal populations develop, each with unique genetic alterations and molecular signatures. Exposure to cancer-directed therapies further drives tumor evolution, as some subpopulations of tumor cells are cleared and others demonstrate or develop resistance. During the metastatic process, additional changes may occur during cellular adaptation, leading to temporal heterogeneity as well (Figure 2C).

While intratumoral heterogeneity is a key player in development of treatment resistance to all cancer-directed therapies including ADCs, current clinical management approaches do not thoroughly assess the state of tumor heterogeneity in a patient. Specifically, treatment decisions are frequently made based on a single biopsy specimen or the analysis of one tissue block from a surgical resection specimen. This approach may not accurately capture the heterogeneity underlying a malignancy within a single patient. For example, it has been shown that in NSCLC up to 76% of subclonal mutations could be missed when comparing findings of a single-site biopsy to multiple biopsies from the same patient [43]. As it relates to ADCs, assessment of target antigen expression in a single biopsy specimen is the current clinical method to identify candidates for therapy. This biopsy specimen is most often evaluated through immunohistochemical (IHC) staining and target antigen expression scoring by the reading pathologist. This particularly applies for T-DM1 and MIRV, in which a threshold score is necessary for clinical use of these agents. Higher levels of HER2 and FRα positivity have been correlated with improved therapeutic outcomes in these drugs, respectively [44,45]. Notably, T-DXd is approved for cancers with low-expression of HER2, as opposed to the higher expression required for T-DM1 [20]. There is not a threshold expression level required for the clinical use of the ADCs targeting the other three antigens (Trop2, Nectin-4, or TF).

To further complicate clinical management, ADC target antigen expression is often evaluated based on pathology specimens at the time of diagnosis rather than recurrence when the ADC therapy is being considered. Given the temporal heterogeneity of tumors, target antigen expression levels may have shifted over time, thus altering the response to therapy in the recurrent setting. Thus, a repeat biopsy at the time when an ADC therapy is being considered may provide the best prediction of likelihood of clinical response.

The clinical assessment of intratumoral heterogeneity at multiple tumor sites to obtain a more complete picture of the scope of heterogeneity in a single patient may be beneficial, but is currently limited by cost, bioinformatic computational requirements to integrate the results, and feasibility in each patient. One approach to better characterize ADC target antigen expression across spatial heterogeneity that is currently being investigated is the sampling of multiple regions within a single lesion, analogous to the 12–14 prostate tissue cores that are utilized to assign a Gleason score [37,46]. There are several limitations to this approach, including procedural risks and cost.

Less invasive methods to assess heterogeneity are also under evaluation, such as blood-based liquid biopsies for characterization of circulating tumor cells (CTC) [47] and next generation sequencing of circulating tumor DNA (ctDNA) [37,48]. Liquid biopsies present an opportunity to capture spatial heterogeneity of multiple tumor sites within a patient, giving a more accurate overall assessment of the relative abundance of tumor cell populations [49]. Furthermore, serial plasma sampling during the course of clinical treatments allows characterization of temporal heterogeneity to help guide treatment decisions. Overall, the level of target antigen expression necessary for effective ADC therapy requires further study and subsequent correlation with patient-specific outcomes; these assessments will be greatly facilitated by serial collection of patient biospecimens.

## 4. Next-Generation ADCs

Continued research focuses on optimization of ADC design by addressing the three central components of antibody target, linker, and payload. Furthermore, exploiting the positive aspects of treatment bystander effect to target antigen-negative tumor cells and/or tumor microenvironment (TME) cells has the potential to enhance ADC efficacy.

### 4.1 Novel antibody targets

Multiple new tumor cell antigens are being investigated for ADC development [50,51] with increasingly thorough algorithms to predict optimal surface antigens [52]. For example, top targets under investigation in 2024 included the transmembrane proteins B7-H3 and claudin 18 isoform 2 (CLDN18.2), in addition to HER2 and Trop2 [50]. Additional targets either recently or currently in clinical trial include the human epidermal growth factor receptor (EGFR), human epidermal growth factor receptor 3 (HER3), carcinoembryonic antigen-related cell adhesion molecule 5 (CEACAM5), sodium-dependent phosphate transport protein 2B (SLC34A2), Ephrin-A4 (EFNA4), and c-MET [51]. As with the antigen targets of the currently FDA-approved ADCs, these proteins are all cell surface proteins with functions in cell signaling. The number of antigen targets and clinical trials is increasing dramatically, with almost 90 targets reported [53].

Additional studies are ongoing to evaluate the therapeutic benefit of targeting antigens on cells in the tumor microenvironment instead of tumor cell antigens. For example, an ADC has been developed targeting T regulatory cells (Tregs), to remove these immunosuppressive cells from the TME. This approach has demonstrated efficacy in preclinical studies [54], which has led to the development of a phase I clinical trial using this type of ADC [55].

### 4.2 Dual-target ADCs

Another strategy to increase ADC efficacy and potentially circumvent tumor heterogeneity is the use of dual-target ADCs, which bind to two separate antigens with one drug complex. These ADCs are termed *bispecific* if targeting two different proteins or *biparatropic* if targeting two different epitopes on the same protein. Bispecific ADCs can help overcome tumor heterogeneity by targeting two different proteins that are each expressed on a subset of tumor cells, thus increasing the total percentage of cells targeted. Furthermore, bispecific ADCs targeting antigens involved in complementary signaling pathways may address the resistance mechanism of bypass signaling, as described later.

Dual-target ADCs may also allow for increased internalization and degradation of the complex for improved payload release. For example, preclinical studies demonstrate a superior killing effect with a bispecific ADC targeting both HER2 and prolactin receptor in breast cancer cells that co-express each target due to the enhanced internalization of prolactin receptor [56]. Biparatropic ADCs that target separate epitopes on HER2 have demonstrated benefit in preclinical T-DM1 resistant tumor models by induction of HER2 receptor clustering and enhanced internalization and degradation of the drug complex for improved delivery of the cytotoxic payload [57].

### 4.3 Novel payloads

ADCs can also be further improved through alterations to the payload. Traditional ADCs all employ cytotoxic drugs as their payload, with currently approved solid tumor ADCs all utilizing payloads that target either microtubules or topoisomerase I. Dual-payload ADCs utilizing more than one cytotoxic drug have shown benefit in preclinical studies [58,59], with two agents now entering phase 1 trials listed on clinicaltrials.gov. Modifying the chemical structure of payloads may decrease vulnerability to drug efflux pumps [39]. Additionally, other cytotoxic drugs are being studied, including the DNA-targeting agents calicheamicin and pyrrolobenzodiazepine (PBD) dimers that are approved payloads for liquid tumor ADCs and the RNA-II polymerase inhibitor alpha amanitin [51].

Non-cytotoxic drug payloads have also been evaluated, such as those employed in immune-stimulating ADCs (iADCs). iADCs utilize a monoclonal antibody targeting a cancer cell surface antigen linked to an immune stimulating agonist, such as toll-like receptor (TLR) or stimulator of interferon genes (STING). TLR drugs have been shown to be too toxic when administered on a systemic level; however, there is promise with the targeted drug delivery provided by antibody conjugation. Based on preclinical studies [60], there are now phase I trials looking at treatment with iADCs [51].

### 4.4 Bystander effect and TME delivery

Optimizing the bystander effect of ADCs has the potential to overcome tumor heterogeneity and successfully clear tumor cells that do not express the target antigen. The degree of bystander effect is impacted by the charge and size of the payload as well as the cleavability of the linker. Preclinical models have demonstrated the presence of bystander effect in multiple currently FDA-approved ADCs [61,62]. It is proposed that bystander effect may help to explain T-DXd efficacy in both HER2-positive and HER2-low or -ultralow breast cancers. Bystander effect can be further optimized with manipulation of the linker and DAR technology in future development of ADCs [39,63]. Another strategy to improve ADC delivery and decrease toxicity is the use of probodies, in which a masked antibody is cleaved by TME proteases or hydrolyzed by the acidity of the TME to then expose the antibody locally and allow target binding [51,64].

## 5. Mechanisms of Resistance to ADCs

Resistance to ADC treatment can either be primary/inherent or secondary/acquired [39]. Primary/inherent resistance can result from insufficient target antigen expression or TME characteristics such as insufficient vascular permeability for ADC delivery [65]. Secondary/acquired resistance develops due to a number of different processes attributable to the unique structure and mechanism of ADCs. For example, resistance can develop either based on changes in antibody targeting or due to alterations to the efficacy of the cytotoxic payload.

Resistance can develop due to the decreased ability of the antibody moiety of the ADC to target the tumor cells. These alterations can include target antigen receptor shedding, upregulation of alternate surface receptors, or bypass signaling [66]. Bypass signaling refers to the preferential transition to another signaling pathway to achieve the same intended effect of the pathway involved by the target antigen [67]. One example of an alteration in antibody efficacy is resistance to trastuzumab via the truncation of the HER2 ectodomain; this could also occur with HER2-targeting ADCs [39].

Once an ADC has been successfully delivered to its target and internalized, there are multiple intracellular mechanisms by which resistance may develop. First, there can be aberrant lysosomal processing of the ADC due to increased lysosomal pH or altered trafficking of the complex within the cytoplasm [38,39]. ADCs with non-cleavable linkers are particularly susceptible to triggering resistance based on defective processing, thus favoring the development of cleavable linkers. Once the payload is separated from the drug complex, there are multiple potential mechanisms of resistance to the payload. These include increased drug efflux via ATP-binding cassette transporters, development of compensatory anti-apoptotic or pro-growth alterations, or changes in the payload drug target [39].

Not only does intratumoral heterogeneity influence primary efficacy and resistance to ADC therapy, but the development of secondary resistance is also affected by heterogeneity with the potential for different resistance mechanisms to arise within different tumor subpopulations within a single tumor site or patient [38,68]. Proteomics studies into mechanisms of resistance show that multiple mechanisms are likely to develop before one ends up predominating [38]. It is difficult to predict the specific mechanism of resistance that will develop within an individual patient, and these efforts are further hindered by the lack of routine post-treatment biopsy sampling. Current research is ongoing to identify early biomarkers that may predict response and resistance [69]. Furthermore, improved preclinical models must be created that recapitulate the tumor heterogeneity seen in patients to best define and characterize the impact of these multiple cell populations on treatment efficacy and resistance.

## 6. Combining ADCs with a Second Therapy

**Figure 3.**
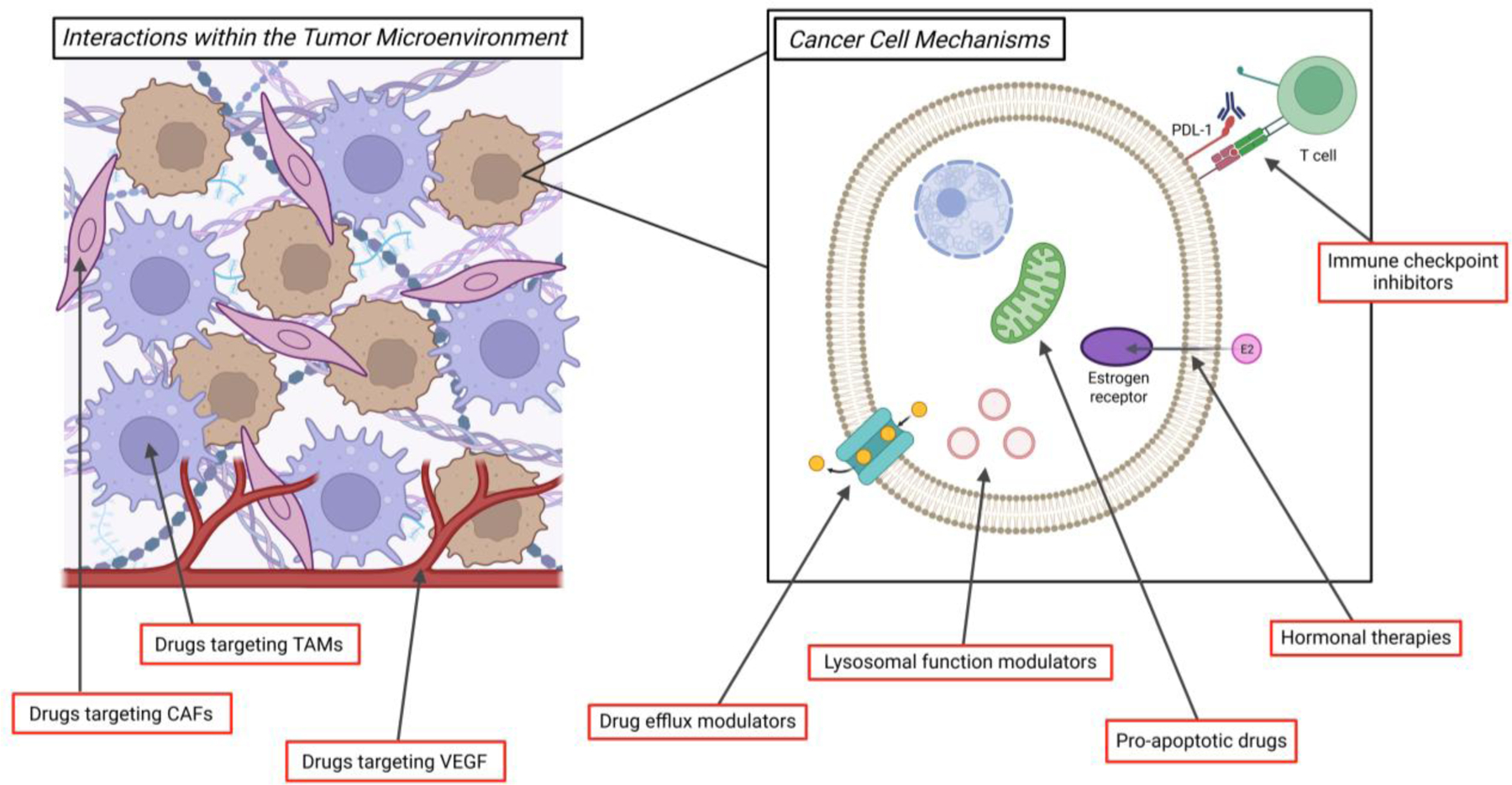
Options for Combinatorial Therapy with ADCs. This figure illustrates potential targets within the TME and the tumor cancer cell to consider for drugs used in combination with ADCs. This figure was created using Biorender.com.

Despite the clinical successes of ADCs to date, tumor heterogeneity and acquired resistance remain major obstacles to achieving a durable response. One fundamental approach of cancer therapy that is being utilized to address these challenges across tumor types is to use combinatorial treatments. This is being attempted within the context of a single ADC agent, using the aforementioned strategies of dual-payload ADCS or dual-target ADCs, which bind to two separate antigens. Furthermore, treatment strategies that employ two different ADCs administered either simultaneously or sequentially may help overcome the heterogeneity of target antigen expression or payload-specific resistance within the tumor. In this section, we will discuss in further detail multiple non-ADC second agents that are currently under preclinical and clinical investigation in combination with ADCs, with the goals of optimizing efficacy and limiting toxicity (Figure 3).

### 6.1 Combinatorial therapies stimulating immune responses

Approaches combining ADCs with immunomodulatory agents are of significant interest. In preclinical studies, administration of an ADC plus an immune checkpoint inhibitor (ICI), such as anti-programmed cell death protein 1 (PD-1)/programmed death-ligand 1 (PD-L1) or anti-cytotoxic T-lymphocyte-associated protein 4 (CTLA4) therapies, results in enhanced immunogenic cell death (ICD) [70,71]. Clinical trials combining ADCs with ICI therapy have yielded mixed results [72,73], thus ongoing research is necessary.

Another strategy is to combine ADCs with therapies targeting immune cells in the TME, such as tumor associated macrophages (TAMs). CD47 is expressed on tumor cells and facilitates macrophage immune evasion [74]. CD47 blockade has been found to enhance the response of breast cancer cells to T-DXd in a preclinical model [62]. TAMs promote ADC resistance by multiple mechanisms including antigen shedding and ADC degradation within the TME [70]. Inhibition of the pro-TAM factor colony stimulating factor 1 receptor (CSF1R) has been studied with a number of small molecule targeted therapies and may have potential as a combinatorial therapy with ADCs [75].

### 6.2 Non-immune combinatorial targets within the TME

There are multiple additional potential targets within the TME for consideration as combinatorial therapies with ADCs. One known successful target is vascular endothelial growth factor (VEGF), for which there are multiple therapeutic options including the monoclonal antibody bevacizumab. It is hypothesized that antiangiogenic agents like bevacizumab may help to normalize tumor vasculature and improve ADC delivery to the target tumor cells. This combinatorial approach has been successfully employed with mirvetuximab soravtansine plus bevacizumab in platinum-resistant ovarian cancers, with a lower tumor FRα antigen threshold expression level needed for the combination of the two drugs than that required for single agent mirvetuximab soravtansine [76].

Proteases within the TME are another potential target for combinatorial therapy, with the goal of blocking protease-mediated release of an ADC’s cytotoxic payload prior to reaching its target cell. Inhibition of the cathepsin L protease has been proposed as a treatment for combination with ADCs to allow for a more targeted cytotoxic payload effect [70]. However, the complexity of combinatorial approaches for ADC treatment is highlighted by the fact that the functional activity of cathepsin L is necessary in HER2 low breast cancer models for effective treatment with T-DXd [62]. Finally, stromal cells within the TME, including cancer-associated fibroblasts (CAFs), are a potential target due to their role in development of a dense extracellular matrix (ECM) that inhibits ADC penetration [70]. Further investigation is needed into the potential impact of combining ADC treatment with ECM-modifying agents [70].

### 6.3 Additional combinatorial approaches

The option to combine ADCs with endocrine therapy is appealing for the treatment of hormone-sensitive cancers, such as estrogen receptor positive breast cancers and ovarian cancers. Multiple prior and current clinical trials are designed to assess the effectiveness of this combination [77]. Given the unique mechanisms of action of ADCs and endocrine therapies, this treatment strategy may help overcome the tumor heterogeneity that contributes to resistance. Furthermore, the side-effect profile of endocrine therapies is both limited and unique from that of ADCs, helping prevent increased toxicity with combination therapy. Additional potential combination therapies with ADCs include lysosomal function modulators, inhibitors of drug efflux transporters to improve intracellular payload concentrations, and therapies targeting apoptotic cellular processes [70].

## 7. Conclusions

ADCs are favorably changing the landscape of cancer treatment for solid tumors and improving patient outcomes. The unique mechanistic aspects of ADCs in which an antibody targets antigen-expressing tumor cells and the cytotoxic payload is released following tumor cell internalization provide the potential for higher local drug concentrations and decreased systemic toxicity. Continued development and optimization of ADC structure is rapidly moving the field forward. Unfortunately, as with other cancer therapies, tumor heterogeneity creates both inherent and acquired resistance that must be overcome for durable clinical effects. Multiple resistance mechanisms unique to ADCs are being unveiled with their continued preclinical and clinical study and use. Thus, we present here approaches to characterize tumor heterogeneity both before and after treatment as well as strategies for ADC design and combinatorial therapies to attempt to address the unique challenges of heterogeneity to achieve more durable treatment responses. Increasing utilization of plasma-based ctDNA testing will help define correlations between tumor heterogeneity and response to therapy, providing critical insights into optimal therapeutic approaches. While ADCs have demonstrated both benefits and limitations, they are an exciting new option in the toolbox of oncologists and their utility will continue to be driven forward by further preclinical development and clinical trial assessment.

## Figures and Tables

**Table 1:** Current FDA-approved ADCs for solid tumors, listed by year of approval

ADC	Approval	Generation	Antibody Target	Payload	Payload Target	Linker	Cleavable
Trastuzumab emtansine	2013	2^nd^	HER2/ERBB2	DM1 (mertansine)	Microtubules	SMCC	No
Trastuzumab deruxtecan	2019	3^rd^	HER2/ERBB2	DXd (exatecan derivative)	TOPI	tetrapeptide	Yes
Enfortumab vedotin	2019	3^rd^	Nectin-4/PVRL4	MMAE (monomethyl auristatin E)	Microtubules	mc-VC-PABC	Yes
Sacituzumab vedotin	2020	3^rd^	Trop2/TaCSTD2	SN-38 (irinotecan metabolite)	TOPI	CL2A	Yes
Tisotumab vedotin	2021	3^rd^	TF	MMAE (monomethyl auristatin E)	Microtubules	mc-VC-PABC	Yes
Mirvetuximab soravtansine	2022	3^rd^	FRα	DM4 (ravtansine)	Microtubules	sulfo-SPDB	Yes
Datopotamab deruxtecan	2025	3^rd^	Trop2/TACSTD2	DXd (exatecan derivative)	TOPI	tetrapeptide	Yes

## Data Availability

Not applicable.

## Abbreviations

ADC: antibody drug conjugate
CAF: cancer-associated fibroblast
CEACAM5: carcinoembryonic antigen-related cell adhesion molecule 5
CSF1R: colony stimulating factor 1 receptor
ctDNA: circulating tumor DNA
CTC: circulating tumor cell
CTLA4: cytotoxic T-lymphocyte-associated protein 4
DAR: drug-to-antibody ratio
Dato-DXd: datopotamab deruxtecan
ECM: extracellular matrix
EFNA4: ephrin-A4
EGFR: epidermal growth factor receptor
EV: enfortumab vedotin
FDA: Food and Drug Administration
FRα: folate receptor alpha
GPI: glycophosphatidylinositol
HER2: human epidermal growth factor receptor 2
HER3: human epidermal growth factor receptor 3
iADC: immune-stimulating ADC
ICD: immunogenic cell death
ICI: immune checkpoint inhibitor
IHC: immunohistochemical
MAPK: mitogen-activated protein kinase
MIRV: mirvetuximab soravtansine
MMAE: monomethyl auristatin E
NSCLC: non-small cell lung cancer
PBD: pyrrolobenzodiazepine
PD-1: programmed cell death protein 1
PD-L1: programmed death-ligand 1
PI3K/AKT: phosphatidylinositol 3-kinase/protein kinase B
PVRL4: nectin-4
RTK: receptor tyrosine kinase
SG: sacituzumab govitecan
SLC34A2: sodium-dependent phosphate transport protein 2B
STING: stimulator of interferon genes
TACSTD2: tumor-associated calcium signal transducer-2
TAM: tumor associated macrophage
T-DM1: trastuzumab emtansine
T-DXd: trastuzumab deruxtecan
TF: tissue factor
TLR: toll-like receptor
TME: tumor microenvironment
TOPI: topoisomerase
Tregs: T regulatory cells
Trop2: trophoblast cell surface antigen 2
TV: tisotumab vedotin
VEGF: vascular endothelial growth factor
